# Low Muscle Strength Is Associated with Metabolic Risk Factors in Colombian Children: The ACFIES Study

**DOI:** 10.1371/journal.pone.0093150

**Published:** 2014-04-08

**Authors:** Daniel Dylan Cohen, Diego Gómez-Arbeláez, Paul Anthony Camacho, Sandra Pinzon, Claudia Hormiga, Juanita Trejos-Suarez, John Duperly, Patricio Lopez-Jaramillo

**Affiliations:** 1 Dirección de Investigaciones, Fundación Oftalmológica de Santander (FOSCAL), Floridablanca, Santander, Colombia; 2 Facultad de la Ciencias de la Salud, Universidad de Santander (UDES), Bucaramanga, Santander, Colombia; 3 Facultad de Medicina, Universidad de Los Andes, Bogota, Colombia; Universidad Pablo de Olavide, Centro Andaluz de Biología del Desarrollo-CSIC, Spain

## Abstract

**Purpose:**

In youth, poor cardiorespiratory and muscular strength are associated with elevated metabolic risk factors. However, studies examining associations between strength and risk factors have been done exclusively in high income countries, and largely in Caucasian cohorts. The aim of this study was to assess these interactions in schoolchildren in Colombia, a middle income Latin American country.

**Methods:**

We measured body mass index, body composition, handgrip strength (HG), cardiorespiratory fitness (CRF) and metabolic risk factors in 669 low-middle socioeconomic status Colombian schoolchildren (mean age 11.52±1.13, 47% female). Associations between HG, CRF and metabolic risk factors were evaluated.

**Results:**

HG and CRF were inversely associated with blood pressure, HOMA index and a composite metabolic risk score (p<0.001 for all) and HG was also inversely associated with triglycerides and C-reactive protein (CRP) (both p<0.05). Associations between HG and risk factors were marginally weakened after adjusting for CRF, while associations between CRF and these factors were substantially weakened after adjusting for HG. Linear regression analyses showed inverse associations between HG and systolic BP (β = −0.101; p = 0.047), diastolic BP (β = −0.241; p> = 0.001), HOMA (β = −0.164; p = 0.005), triglycerides (β = −0.583; p = 0.026) and CRP (β = −0.183; p = 0.037) but not glucose (p = 0.698) or HDL cholesterol (p = 0.132). The odds ratios for having clustered risk in the weakest quartile compared with the strongest quartile were 3.0 (95% confidence interval: 1.81–4.95).

**Conclusions:**

In Colombian schoolchildren both poorer handgrip strength/kg body mass and cardiorespiratory fitness were associated with a worse metabolic risk profile. Associations were stronger and more consistent between handgrip and risk factors than between cardiorespiratory fitness and these risk factors. Our findings indicate the addition of handgrip dynamometry to non-invasive youth health surveillance programs would improve the accuracy of the assessment of cardio-metabolic health.

## Introduction

In youth, poor performance in measures of cardiorespiratory and muscular fitness are associated with elevated clustered metabolic risk and cardiovascular risk factors such as triglycerides, HOMA index and inflammatory markers [1]–[4]. Fitness tracks from youth into adulthood [5], where both low cardiorespiratory fitness (CRF) and muscular strength are associated with elevated risk of cardiovascular (CV) disease and total mortality [6] ^.^ Evidence that both of these components of fitness are declining in children internationally [7], [8] is therefore worrying from a public health perspective. Based on associations with health and the feasibility of implementing these measures on a large scale in schools [9], [10], it is argued that the assessment of CRF and muscular strength should be included as part of health monitoring in youth [11]. Nonetheless, to justify their inclusion in surveillance efforts, particularly in countries where resources are limited, these tests must be validated as markers of CV risk in diverse populations. While associations between CRF and CV risk factors have been evaluated in youth from both high- and low-middle income countries [1]–[4], [12], [13], studies examining interactions between muscular strength and risk factors have been done exclusively in high income countries, and largely in Caucasian cohorts [14]. There is a need to also assess these associations in low-middle income countries, where there is a larger and more rapidly increasing burden of non-communicable disease [15].

The primary aim of this study was to assess relationships between muscular strength, CRF and cardio-metabolic health profile in schoolchildren in Colombia, a middle income Latin American country.

## Materials and Methods

### Study Population

During the 2011–2012 school year, we conducted the cross-sectional component of the ACFIES study (Association between Cardiorespiratory Fitness, Muscular Strength and Body Composition with Metabolic Risk Factors in Colombian Children). The sample comprised 669 low-middle socioeconomic status (SES 1–3 on a scale of 1–6 defined by the Colombian government) schoolchildren (aged 8 to 14 years, 47% female) enrolled in public elementary and high schools (grades 5 and 6) in the city of Bucaramanga, Colombia.

### Ethics Statement

The health research ethics board of the Ophthalmological Foundation of Santander approved all study procedures. The children expressed their interest in participating in the study, and parents or legal guardians gave written informed consent before the children were included in the study.

### Measures

Body weight was measured in underwear and no shoes, using electronic scales (Tanita BC544, Tokyo, Japan). Height was measured using a mechanical stadiometer platform (Seca 274, Hamburg, Germany). Body mass index (BMI) was calculated by dividing body weight (kg) by height squared (cm^2^). Waist circumference was measured at the midpoint between the last rib and the iliac crest using a tape measure (Ohaus 8004-MA, NJ, USA). Body composition was estimated using bioelectrical impedance analysis (Tanita BC544, Tokyo, Japan). Blood pressure was assessed after a period of sitting using an automatic oscillometric device (Omron HEM 757 CAN, Hoofddorp, Holland) with a pediatric cuff. Sexual maturation was determined by stage of secondary sexual development by a physician following the methodology described by Tanner and Whitehouse [16].

Blood samples of 20 ml were taken from the antecubital vein between 07∶00 and 09∶00 am after an overnight fast. Participants were asked not to do any prolonged exercise in the previous 24 hours. The tests were processed in the laboratory of the School of Bacteriology, University of Santander. The glucose and lipid profile were assessed using a routine colorimetric method (BTS-303 Biosystem photometric, Barcelona, Spain). hsCRP was quantified using a turbid metric test (SPINREACT, Spain). Insulin levels were determined using an insulin microplate ELISA test (Monobind, USA).

### Metabolic Risk

We calculated a continuous composite metabolic risk score (MRS) by summing standardized residuals (z-score) by age and sex for HOMA score, waist circumference, TG, HDL-c, and systolic blood pressure. These variables are used as criteria for the metabolic syndrome in adults [17] and youth [18]. For calculation of odds ratio for elevated metabolic risk score - “clustered risk” - was defined as 1 SD above the age and sex specific mean, a cut-point used previously in similar studies in youth [1], [2].

### Physical Fitness and Physical Activity

Aerobic Capacity was evaluated in groups using a maximum incremental indirect field test [19], as previously reported [20]. In brief, the test requires participants to run “shuttles” back and forth between two lines 20 m apart, in time with an audible “bleep” signal starting at an initial speed of 8.0 km.^h–1^, a speed which is a brisk walk or slow jog for most children. After one minute of shuttles at this pace the audible signal changes to alert participants to the beginning of the next “level” and an increase in speed. Participants were encouraged to run for as long as possible, but allowed to ‘drop out’ of the test at any time if they felt unable to continue or to maintain the pace. They were also told that their test would be terminated if they failed to reach the line in time for the bleep on two consecutive shuttles.

Muscular strength was measured with a Takei analogue handgrip dynamometer (Takei T.K.K.5001 Grip A Dynamometer, Takei Scientific Instruments Co. Ltd. Niigata-City, Japan). Pupils were given a brief demonstration and verbal instructions for the test and if necessary the dynamometer was adjusted according to the child’s hand size. The test was done in the standing position with the wrist in the neutral position and the elbow extended children were given verbal encouragement to ‘squeeze as hard as possible’ and apply maximal effort for at least 2 seconds. Two trials were allowed in each limb and the highest score recorded as peak grip strength (kg). To account for differences in body size, peak grip strength was divided by body mass and HG/kg was used in further analysis. Children also completed the PAQ-C physical activity questionnaire, a 7-day recall tool which provides an estimate of overall physical activity based on self-reported sporting activities, school lunchtimes, physical education classes as well as after school and weekend activity and has reasonable validity in this age group [21].

### Statistical Analysis

Analysis of the variables and measures studied was done using STATA (version 11.2, StataCorp 2009. Stata Statistical Software: Release 11. College Station, TX: StataCorp LP). Means and SD by sex for anthropometric variables, fitness and blood measures were calculated. For analysis of difference by sex, student t test or Mann Whitney test were used for continuous variables and chi-square for categorical variables. We estimated the partial correlations between handgrip strength or cardiorespiratory fitness and individual CV and metabolic risk factors and MRS, adjusted for sex, age and pubertal stage. To assess the independence of the associations between the two types of fitness and metabolic risk factors we adjusted handgrip strength for CRF in a second model and in a third model, CRF was adjusted for handgrip strength. To evaluate differences in metabolic risk across quartiles of handgrip ANOVA was used with analysis of linear trend using Kruskal–Wallis tests. We further examined the association between handgrip strength and individual risk factors and MRS using robust multiple linear regression with HG adjusted for age, sex, maturation and CRF in all participants and then separately in children dichotomized by %body fat. We divided participants into normal fat (1^st^ and 2^nd^ third of % body fat) and high fat (upper 3^rd^ of body fat %) groups. Robust logistic regression was then used to estimate odds ratios for risk of having clustered metabolic risk according to quartile of handgrip strength in the whole group and then in boys and girls separately.

## Results

Descriptive statistics for girls and boys are shown in table 1. Absolute handgrip strength was not significantly difference between boy and girls but boys had significantly higher scores in handgrip/kg body mass (p = 0.003), cardiorespiratory fitness (p<0.001) and self-report physical activity level (p<0.001). Girls had significantly higher % body fat but boys had significantly higher WC (p = 0.005), BMI and significantly fewer were classified as normal weight (p = 0.041). The prevalence of overweight and obesity did not differ by sex. Boys had significantly higher SBP (p<0.012), girls had significantly higher Insulin (p<0.001), HOMA Index (p<0.002) and triglycerides (p = 0.048) but composite metabolic risk score was not significantly different (p = 0.708). There was no significant difference in anthropometric or fitness variables between the 546 children with blood measures compared to the 123 who did not.

**Table 1:** Characteristics of population.

	Total (n = 669)	Girls (n = 318)	Boys (n = 351)	p value
Age (years)	11.52±1.13	11.52±1.10	11.51±1.16	0.838
Body mass (kg)	40.08±10.07	40.33±9.77	39.86±10.35	0.636
Height (m)	1.45±0.09	1.46±0.08	1.44±0.09	0.339
BMI (kg/m^2^)	18.87±3.61	18.81±3.52	18.93±3.68	0.004
Waist circumference (cm)	65.95±9.73	64.86±9.02	66.92±10.24	0.005
Tanner stage n (%)*				
1	368 (56.3)	149 (47.8)	219 (62.0)	0.004
2	208 (31.8)	110 (35.3)	98 (28.7)	0.308
3	78 (11.9)	53 (16.9)	25 (7.3)	0.249
Weight status n (%)**				
Underweight	29 (4.41)	9 (2.9)	20 (5.8)	0.739
Normal weight	479 (72.8)	240 (77.2)	239 (68.9)	0.041
Overweight	85 (12.9)	42 (13.5)	43 (12.4)	0.879
Obese	65 (9.8)	20 (6.4)	45 (12.9)	0.436
% body fat	20.47 (7.50)	22.71 (6.89)	18.43 (7.46)	<0.001
Handgrip (kg)	15.99±4.36	15.61±3.74	16.32±4.83	0.256
Handgrip (kg)/kg body mass	0.41±0.09	0.39±0.08	0.42±0.09	0.003
CRF (# of shuttles)	25.99±14.09	22.6±10.51	28.99±16.05	<0.001
PAQ-C score	2.77±0.59	2.64±0.57	2.88±0.58	<0.001
SBP (mmHg)	114.51±11.59	113.29±11.72	115.58±11.38	0.012
DBP (mmHg)	73.78±9.47	73.66±8.97	73.86±9.93	0.994
Glucose (mg/dl) β	88.52±12.56	87.87±12.32	89.12±12.76	0.216
Insulin (Ul/ml)	2.58±2.61	2.91±2.91	2.29±2.26	<0.001
HOMA index	0.57±0.58	0.64±0.66	0.49±0.49	0.002
HDL-C (mg/dl)	75.34±19.96	74.57±19.81	76.04±20.08	0.411
Triglycerides (mg/dl)	91.76±52.37	94.07±46.79	89.66±56.97	0.048
CRP (mg/dl)	0.89±1.62	0.88±1.52	0.89±1.71	0.391
Metabolic risk score	−.055±2.93	−0.042±2.82	−0.066±3.04	0.708

Mean ± standard deviation, except weight status (%). Differences between boys and girls calculated using Wilcoxon rank-sum test, except for glucose using ANOVA and weight status (χ^2^ test). BMI, body mass index; Weight status, classified according to Barlow [22]; CRF, Cardiorespiratory fitness; PAQ-C score, Physical activity questionnaire; SBP, systolic blood pressure; DBP, diastolic blood pressure; HOMA index, homeostatic model assessment index; HDL, High Density Lipoprotein; TC/cHDL, Total cholesterol/High Density Lipoprotein; CRP, C-Reactive Protein.

*data missing for 11 participants.

**data missing for 15 participants.

**Figure 1:**
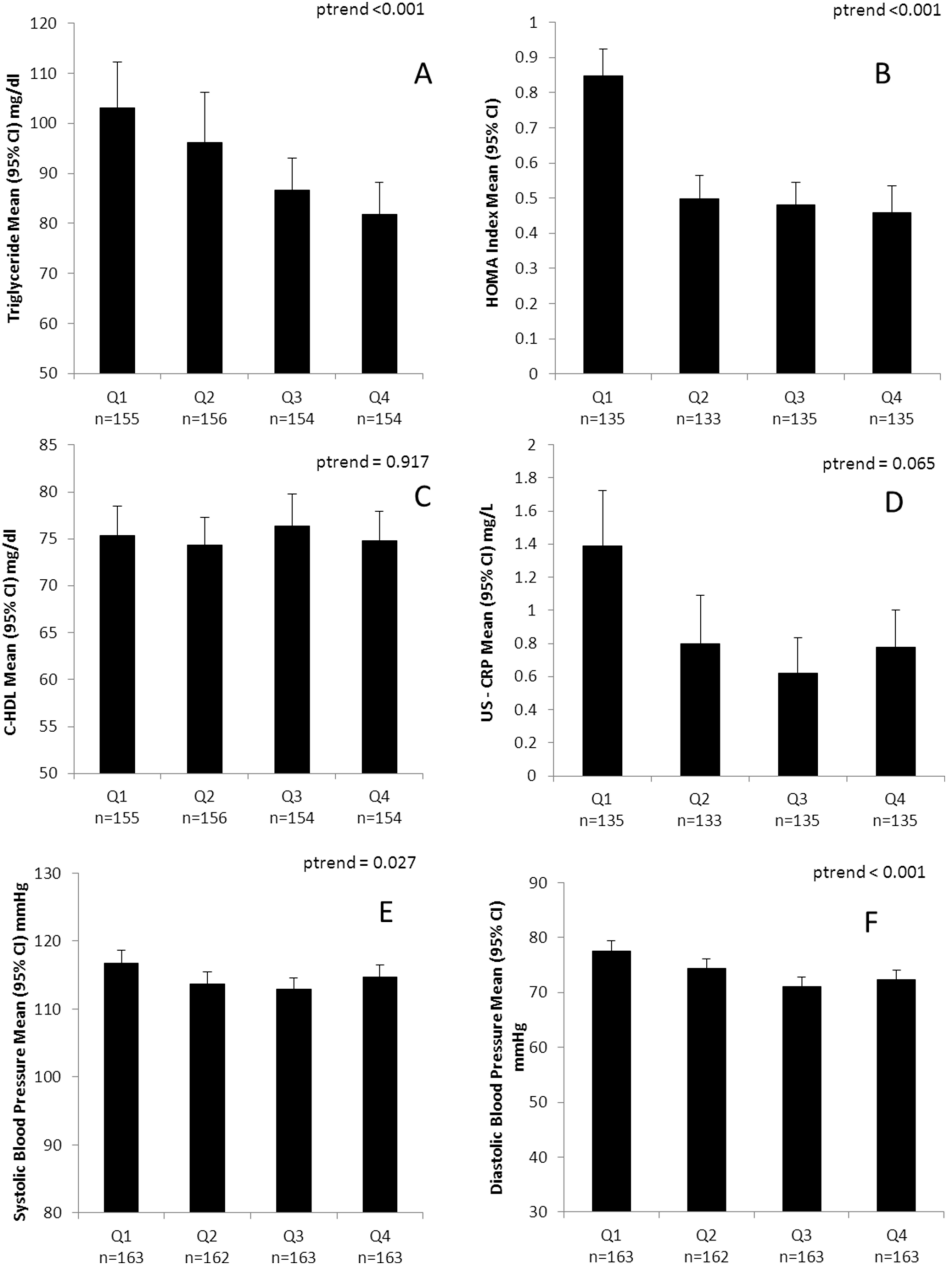
Metabolic risk factors according to quartile of handgrip strength in 8–14 year old children (n = 536). Quartile 4 is the highest HG category. Handgrip quartiles are age and sex specific handgrip/kg body mass. Linear trend evaluated using Kruskal–Wallis test.

**Figure 2:**
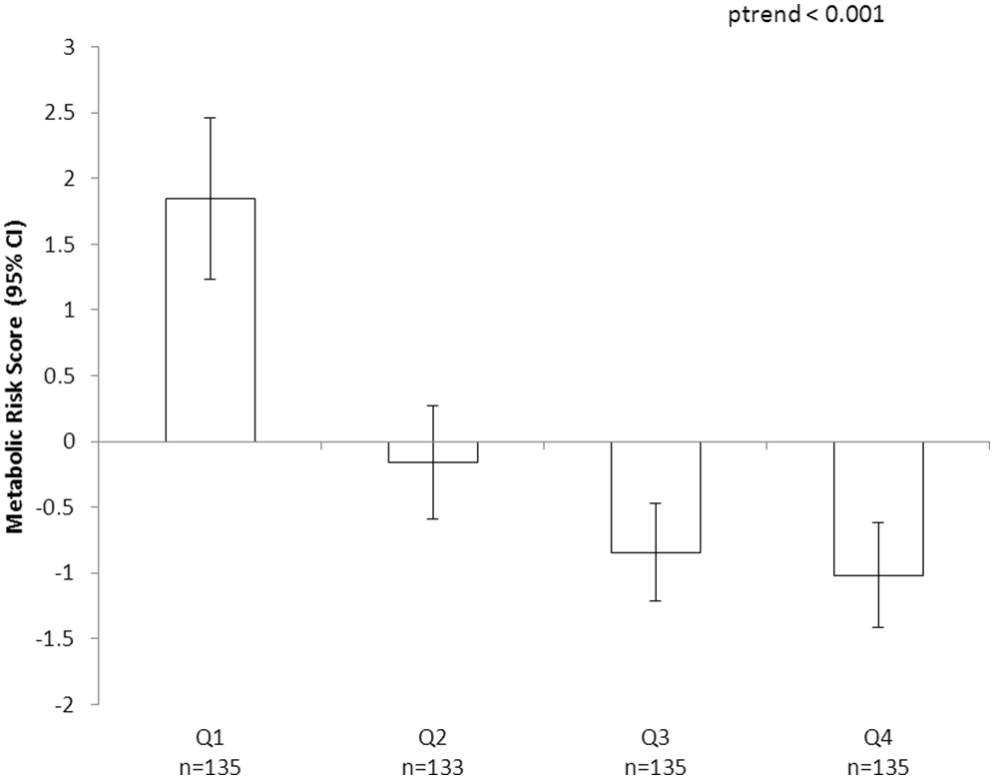
Metabolic risk score according to quartile of handgrip strength in 8–14 year old children (n = 536). Quartile 4 is the highest HG category. Handgrip quartiles are age and sex specific handgrip/kg body mass. Linear trend evaluated using Kruskal–Wallis test. Metabolic risk score is summed standardized residuals (z-score) by age and sex for HOMA score, waist circumference, TG, HDL-c, and systolic blood pressure.

Partial correlations between handgrip strength (HG), cardiorespiratory fitness (CRF) and CVD risk factors are shown in Table 2. In model 1, adjusted for age, sex and maturation status and physical activity both HG and CRF were inversely associated with BP, HOMA index and metabolic risk score (p<0.001 for all associations). HG but not CRF was also inversely associated with triglycerides and CRP (both p<0.05). After additionally adjusting for CRF (model 2), associations between HG and individuals markers and metabolic risk score (MRS) were only marginally weakened and all remained significant (p<0.001 for DBP and MRS; p<0.05 for all other associations). Associations between CRF and DBP, HOMA and MRS were substantially weakened after adjusting for HG (model 3) but remained significant (p<0.05 for all). Due to the stronger and more robust associations between HG and metabolic risk compared to CRF in the partial correlations, further exploration of interactions were conducted with HG only. Individual risk factors and MRS across age and sex-specific HG quartiles are shown in Figure 1 and Figure 2, respectively. We found significant negative linear trends for HG and triglycerides (p<0.001), HOMA index (p<0.001), SBP (p = 0.027) and DBP (p<0.001) and MRS (p<0.001), a marginally non-significant (p = 0.065) trend for CRP and there was no association with HDL-C (p = 0.917).

**Table 2:** Partial correlations between handgrip or cardiorespiratory fitness and risk factors.

	Handgrip/kg body mass (HG)	Cardiorespiratory Fitness (CRF)
Model 1:HG and CRF adjusted by sex, age, maturation, PAQ		
log SBP (mmHg)	−0.077	0.0003
log DBP (mmHg)	−0.196**	−0.148**
Glucose	−0.028	−0.039
Log HOMA index	−0.1775**	−0.162**
Log Triglycerides	−0.104*	−0.037
Log CRP	−0.107*	−0.045
Log HDL-C	0.016	−0.059
Metabolic Risk Score	−0.357**	−0.2189**
**Model 2: HG adjusted for CRF and by sex, age, maturation, PAQ**		
log SBP (mmHg)	−0.083*	
log DBP (mmHg)	−0.153**	
Glucose	−0.016	
Log HOMA index	−0.127*	
Log Triglycerides	−0.095*	
Log CRP	−0.097*	
Log HDL-C	0.036	
Metabolic Risk Score	−0.298*	
**Model 3: CRF adjusted for HG and by sex, age, maturation, PAQ**		
log SBP (mmHg)		0.031
log DBP (mmHg)		−0.085*
Glucose		−0.027
Log HOMA index		−0.109*
Log Triglycerides		−0.0002
Log CRP		−0.008
Log HDL-C		−0.065
Metabolic Risk Score		−0.115*

Handgrip (HG) is handgrip (kg) adjusted for body mass. Cardiorespiratory fitness (CRF) is number of 20 m shuttles.

*p<0.05;

**p<0.001.

### Interactions between Handgrip Strength, Body Composition and Risk Factors

Linear regression analyses with HG adjusted for age, sex, pubertal stage and CRF (Table 3) revealed inverse associations with SBP (β = −0.101; p = 0.047), DBP (β = −0.241; p<0.001), HOMA (β = −0.164; p = 0.005), triglycerides (β = −0.583; p = 0.026), CRP (β = −0.183; p = 0.037) and MRS (β = −10.34; p<0.001) but not glucose (p = 0.698) or HDL cholesterol (p = 0.132). The same regression analysis was applied to the group dichotomized into normal or high % body fat groups. In the high fat group, HG was inversely associated with HOMA index (p = 0.027), CRP (p = 0.011), SBP (p = 0.022) and DBP (p = 0.004) and MRS (p<0.001), but there were no significant associations between HG and risk factors in the normal fat group.

**Table 3:** Association between handgrip strength/kg body mass and metabolic risk factors in 8–14 year old children.

	*All*			*T1–T2% body fat*			*T3% body fat*		
	β coeff	SE	p value	β coeff	SE	p value	β coeff	SE	p value
**Log Systolic Blood Pressure**	−0.101	0.051	**0.047**	0.069	0.061	0.251	−0.134	0.106	0.208
**Log Diastolic Blood Pressure**	−0.241	0.064	**<0.001**	0.054	0.078	0.485	−0.378	0.128	**0.004**
**Glucose**	−2.471	6.355	0.698	3.458	8.462	0.683	−1.466	13.65	0.915
**Log HOMA Index**	−1.644	0.579	**0.005**	−0.010	0.743	0.989	−2.507	1.126	**0.027**
**Log Triglycerides**	−0.583	0.261	**0.026**	−0.271	0.308	0.380	0.315	0.554	0.570
**Log CRP**	−1.833	0.879	**0.037**	−0.243	1.017	0.811	−4.981	1.931	**0.011**
**log HDL- C**	0.117	0.131	0.370	0.061	0.165	0.714	−0.097	0.293	0.740
**Metabolic Risk Score**	−10.34	1.628	**<0.001**	−1.782	1.481	0.245	−14.754	3.468	**<0.001**

Linear regression analysis adjusted for sex, age, tanner and cardiorespiratory fitness. T1–T2 = Low and middle third of % body fat; T3 = Upper third of % body fat.

**Figure 3:**
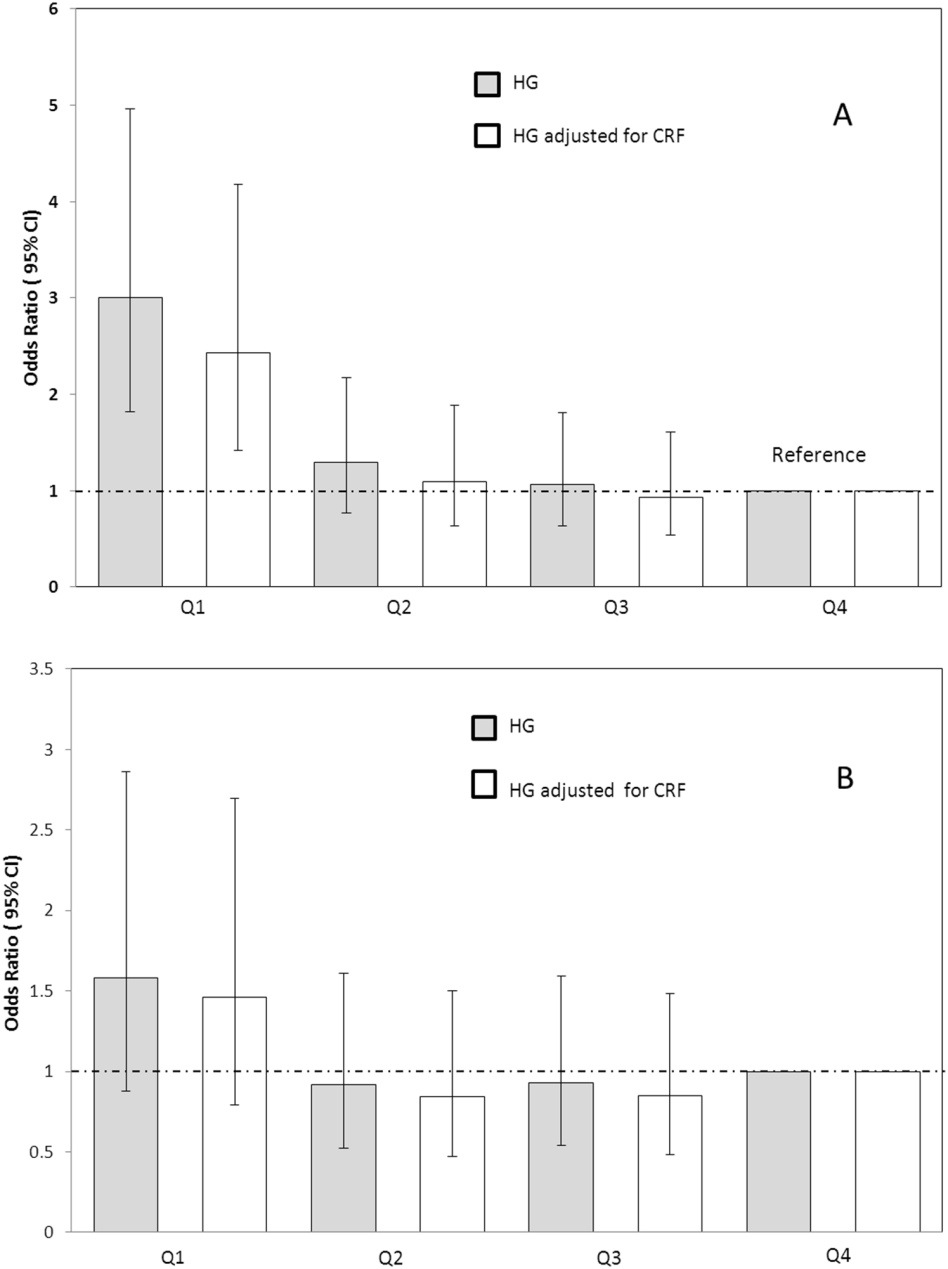
Handgrip strength quartiles and risk of clustered metabolic risk in 8–14 year old children (n = 536). Quartile 4 is highest handgrip strength category (used as referent). Handgrip quartiles are age and sex specific handgrip/kg body mass unadjusted (grey bars) or adjusted for cardiorespiratory fitness (white bars). Clustered risk is defined as 1 SD above the age and sex specific mean. Panel A: Adjusted for Odds ratios (95% CI) for clustered metabolic risk by HG across quartiles in 8–14 year olds (n = 546) age, sex, and maturation status. Panel B: Additionally adjusted for % body fat category.

**Figure 4:**
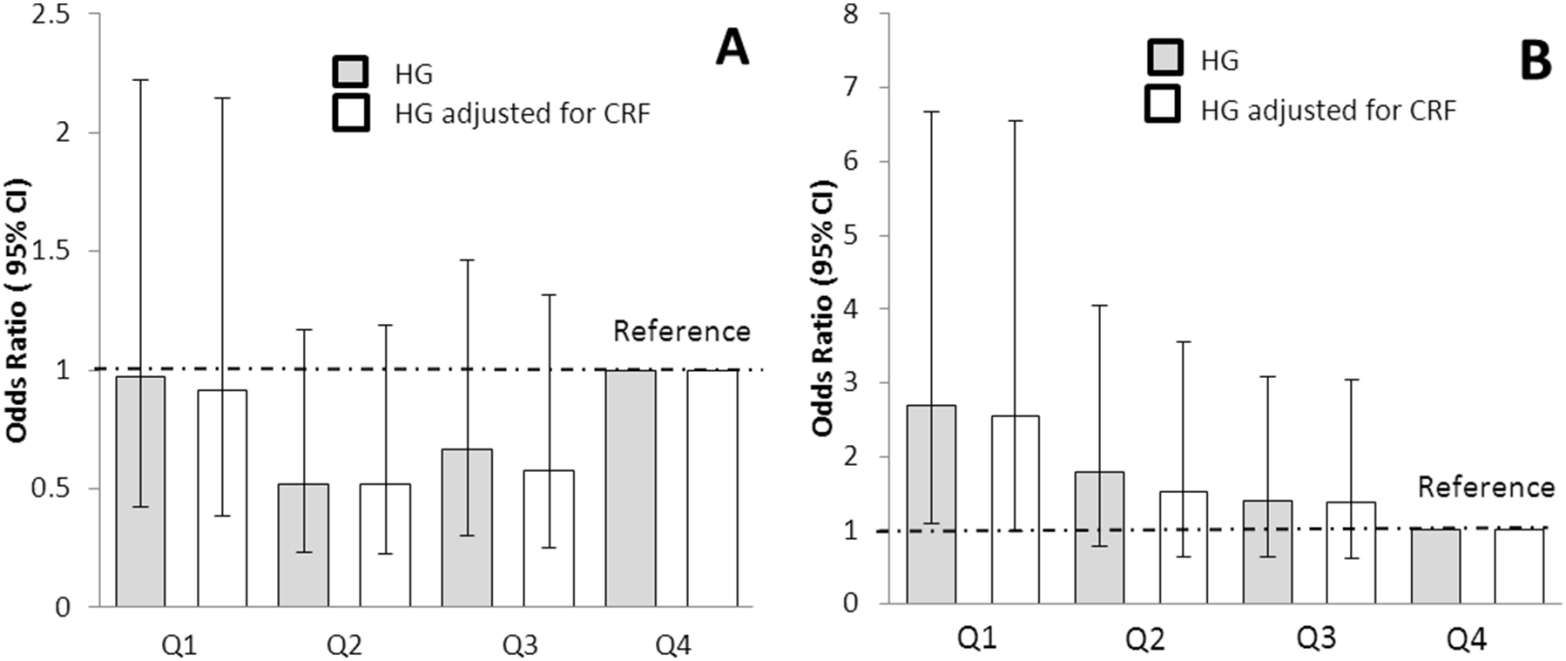
Handgrip strength quartiles and risk of clustered metabolic risk in 8–14 year old girls (Panel A, n = 306) and boys (Panel B, n = 330). Quartile 4 is highest handgrip strength category (used as referent). Adjusted for age, maturation status and % body fat category (grey bars) and additionally for cardiorespiratory fitness (white bars). Clustered risk is defined as 1 SD above the age and sex specific mean.

We then examined the risk of having clustered metabolic risk across quartiles of HG in the weakest fourth (quartile 1) compared with the reference highest strength quartile (quartile 4). The odds ratio (OR) adjusted for age, sex, and maturation status (Figure 3, Panel A) was 3.0 (95% confidence interval (CI), 1.81–4.95) in Q1 compared with Q4, a trend little affected by adjusting for CRF, OR 2.43 (CI 1.41–4.17). After further adjusting for % body fat category (Figure 3, Panel B), the odds ratio for clustered risk in the weakest quartile compared with the strongest (referent) quartile was no longer significant both in CRF-unadjusted OR 1.58 (CI 0.87–2.86) and CRF-adjusted OR 1.45 (CI 0.78–2.69), models. We performed the same regression on the boys and girls separately (Figure 4) and found elevated risk of clustered metabolic risk OR 2.69 (CI 1.1–6.68) in boys (Panel A) in Q1 compared with Q4 but not in girls (panel B) OR 0.97 (CI 0.42–2.22). Again, neither of these trends were substantially affected by adjusting for CRF; in boys OR 2.55 (CI 0.99–6.54) or in girls OR 0.91 (CI 0.38–2.14).

## Discussion

The present study examined interactions between field measures of muscular strength (handgrip strength) and cardiorespiratory fitness (CRF) and metabolic risk factors in Colombian schoolchildren. Handgrip strength (HG) was inversely associated with triglycerides and C-reactive protein, DBP, HOMA index and metabolic risk score and remained so after adjusting for CRF. CRF was also inversely associated with HOMA index, DBP and metabolic risk score but these associations were substantially weakened after adjusting for HG. Youth in the lowest strength quartile were at three times as likely to have elevated metabolic risk than those in the highest strength quartile.

To our knowledge the present study is the first to examine interactions between metabolic risk and both muscular strength and cardiorespiratory fitness in Latin American children or in a pediatric population of a low to middle income country *per se.* This may explain why although our findings broadly confirm previous data in European youth [1]–[4] in showing that both muscular and cardiorespiratory fitness is associated with better cardio-metabolic health; there is a notable difference. We observed stronger associations between HG and cardio-metabolic risk factors than between CRF and these markers, the opposite of the pattern reported in European youth. Partial correlations between HG and risk factors such as HOMA and triglycerides were very similar to these studies [1], [2] but they additionally found inverse associations between CRF and triglycerides and total Cholesterol/HDL-cholesterol, which we did not observe. Moreover, in contrast with our analysis these studies found that adjusting for HG did not substantially weaken relationships between CRF and metabolic risk.

We found negative linear trends across HG-z score quartiles for triglycerides, HOMA index and DBP, CRP and SBP and adjusted for sex, age, maturation and CRF. Regression analysis in the whole group showed inverse associations between HG/kg and SBP, DBP, HOMA index, triglycerides, CRP and metabolic risk score. However, in regression analysis with the group divided by % body fat, associations between HG/kg and HOMA index, CRP, DBP and metabolic risk score were only found in the children and adolescents with higher % body fat (Table 3). This observation is somewhat consistent with previous studies in European schoolchildren in which adiposity was defined by BMI. Ruiz et al [23] found an association between low HG and higher CRP in overweight/obese children but not in the normal weight and Steene-Johannssen et al [2] found a stronger association between strength and metabolic risk in the overweight compared to normal weight.

The similar methodology, age group and sample size as in the previous studies in European children suggest that ethnic and/or regional differences may be underlie the discrepant findings regarding the strength of associations between CRF versus HG and metabolic risk. Indeed, interactions between ethnicity, region and cardiovascular risk factors have been reported in 10–11 year old children [24], [25], but interactions with physical fitness have not been examined. While interactions between muscle strength, CRF and risk factors have not been previously reported in Latin American children, Bergmann et al. [12] did observe strong inverse relationships between CRF and several metabolic risk factors in Brazilian schoolchildren. These associations appear to be more consistent with those reported in the European studies than that of the present study, but the inclusion of private as well as public schoolchildren in the study also suggests that the sample was of higher social status than the present sample. Perhaps the greater associations between muscle strength and metabolic risk compared to CRF may relate more to the poorer social conditions of our sample rather than regional or ethnic differences. Indeed, in a sample of rural 7–15 year olds in Mozambique, a low income country, dos Santos et al. [13] found no association between CRF and a composite metabolic syndrome score. Lower birth weight, a marker of poorer intra-uterine conditions, and inadequate current nutritional status are both more prevalent in lower socio-economic status communities within low-middle income countries such as Colombia. Low birth weight is associated with lower muscle strength and muscle mass [26] and with alterations in muscle morphology [27] but not with independent deficits in CRF [28] or mitochondrial capacity [29]. Indeed, lower birth-weight was associated with significantly lower HG but higher aerobic fitness in Brazilian children [30]. Low birth weight is also associated with impaired PI3/Akt signalling pathway in skeletal muscle, affecting both with insulin resistance and deficits muscle mass/performance and providing a potential link between the two [31].

In Colombian schoolchildren of similar social status as the present sample, Arsenault et al. [32] found that poorer current micronutrient status was associated with worse performance in the standing long jump, a measure of lower body strength. In Brazilian adolescents from an area with low-middle human development index, 64.6% achieved expected standards for CRF, while only 1.5% attained standards for muscle strength/endurance performance [33]. Amongst 4–6 year old Senegalese children, chronic under-nutrition was associated with significantly lower handgrip strength, but endurance test performance did not differ according to chronic nutritional status [34]. Therefore, both poor current or early life nutrition appear to be more consistently associated with persistent deficits in muscle mass and performance than in CRF, which in turn may contribute to the stronger relative diagnostic value of HG in populations such as the present.

In a sub-analysis of the 28% of the present sample for whom birth weight data was available (N = 213), there does appear to be a stronger association between HG/kg and metabolic risk in the low birth weight schoolchildren (<2800 gm at full term; N = 27, r = −0.599, p = 0.002) than in those with normal birth weight (>2800 gm at full term; N = 186, r = −0.383, p<0.001). However, further research is needed to confirm this exploratory analysis in a larger sample and to evaluate interactions between fitness and metabolic risk score in normal versus low birth weight youth or adults. We did not undertake a complete assessment of current nutritional status, but we do have measures of both albumin and ferritin in the majority of the current sample (N = 526). Partial correlations between HG/kg and metabolic risk score were significant across tertiles of serum albumin (T1: r = −0.406, p<0.001; T2: r = −0.400, p<0.001; T3: r = −0.285, p<0.001) and serum ferritin (T1: r = −0.286, p<0.001; T2: r = −0.391, p<0.001; T3: r = −0.408, p<0.001), with little variation in *r* values. While it does not appear that current nutritional status modulates the association between HG and metabolic risk, studies with a more comprehensive evaluation of current nutritional status are needed to more robustly evaluate this hypothesis. International studies that include populations from developed and developing countries are needed to further examine interactions between region/ethnicity, birth weight, nutritional status, fitness and cardio-metabolic risk factors.

### Potential Mechanisms

The mechanisms underlying the association between muscular strength and cardio-metabolic risk in children remain to be established [2]. These benefits may be mediated by associations between strength and CRF [6] or physical activity [35], both well established as factors associated with better cardio-metabolic health [36]. We also found a moderate correlation between CRF and HG/kg (r = 0.42, p<0.001). However, partial correlations between HG/kg and metabolic risk (Table 2) were only marginally affected by adjusting for CRF, suggesting that in the present population the pathways mediating this association are independent from CRF. Physical activity levels does not appear mediate explain findings as HG/kg was not associated with PAQ score (r = 0.071, p = 0.07). This is consistent with a previous study, which found no association between HG/kg and objectively measured moderate-vigorous PA [37]. However, it is also plausible that HG is associated with participation in specific sub-types of physical activity or sports that we did not quantify.

A potential explanation for the inverse association between strength and HOMA index in particular, relates to HG/kg a marker of muscle mass. However, while muscle mass was reported to be an independent predictor of insulin sensitivity in normal weight Indian men [38], Lee et al found no association between the two variables in obese boys [39]. Moreover, we found that while raw HG was strongly associated with lean body mass (0.7, p<0.001) HG/kg was not. HG/kg was however positively associated with % lean mass (r = 0.6, p<0.001) (and inversely with % body fat), suggesting that the cardio-metabolic benefits of HG/kg might relate to its association with body composition, rather than muscle mass *per se.* Indeed, in girls adjusting for % body fat category eliminated the elevated risk of clustered metabolic risk associated with low HG/kg (Figure 3, Panel A). However, this was not the case in boys (Figure 3, Panel B), for whom the cardio-metabolic benefits associated with adequate HG/kg are not explained by its associations with body composition. Further work is needed to define the mechanisms by which HG/kg is associated with lower metabolic risk.

### Limitations

Due to the cross-sectional nature of our study, we cannot infer a causal association between low strength and cardio-metabolic risk. Strength training however has been shown to improve components of metabolic risk such as insulin sensitivity [40], [41] in overweight youth, although equivalent studies in normal weight youth or low-middle income populations are lacking. Another potential weakness of our study is that the evaluation of fitness using indirect field measures rather than with “gold standard” laboratory measures such as one repetition maximum strength testing or assessment of VO_2_ max for CRF. Nonetheless, the field measures we used are reliable and valid [42], were used in previous studies of these interactions [1]–[3] and have the advantage that they can feasibly be implemented on a large scale in schools [10], [20]. Finally, our sample included only social classes one to three on a scale of one to six (one is the lowest) and our findings might not be generalizable to middle-upper class Colombian schoolchildren. Nonetheless, over 85% of Colombians are in social classes 1–3, and is therefore representative of the social conditions under which the large majority of youth in the country live [43].

## Conclusions

Surveillance of chronic NCD risk factors is justified on the basis that trends in modifiable risk factors can predict future disease patterns [15]. Low strength in adolescence associated with poorer current cardio-metabolic health, predicts higher cardiovascular risk in young adulthood [44] and CV and total mortality in adulthood [45]. Based on evidence such as this, it is argued that fitness measures should be included in European youth health surveillance [11]. Our findings suggest that in Latin American children the accuracy of the classification of cardio-metabolic risk profile in non-invasive health surveillance would also be enhanced by inclusion of HG assessment.

The greater diagnostic value of strength compared to cardiorespiratory fitness in this sample deserves attention and should be confirmed in similar populations. Future research should also aim to evaluate whether a greater emphasis on physical activity that develops strength is warranted for youth in low-middle income countries.
